# Complete Genome Sequence of *Caloramator* sp. Strain E03, a Novel Ethanologenic, Thermophilic, Obligately Anaerobic Bacterium

**DOI:** 10.1128/MRA.00708-19

**Published:** 2019-08-08

**Authors:** E. Anne Hatmaker, Dawn M. Klingeman, Roman K. Martin, Adam M. Guss, James G. Elkins

**Affiliations:** a Biosciences Division, Oak Ridge National Laboratory, Oak Ridge, Tennessee, USA; b The Center for Bioenergy Innovation, Oak Ridge National Laboratory, Oak Ridge, Tennessee, USA; c Bredesen Center for Interdisciplinary Research and Graduate Education, University of Tennessee—Knoxville, Knoxville, Tennessee, USA; Indiana University, Bloomington

## Abstract

Here, we report the complete genome sequence of Caloramator sp. strain E03, an anaerobic thermophile that was isolated from a hot spring within the Rabbit Creek area of Yellowstone National Park. The assembly contains a single 2,984,770-bp contig with a G+C content of 31.3% and is predicted to encode 2,678 proteins.

## ANNOUNCEMENT

The genus *Caloramator* is included within the family Clostridiaceae and represents a group of rod-shaped, sporogenic, obligately anaerobic microorganisms that ferment a wide range of substrates at moderately thermophilic temperatures (1). Geographically, the organisms are widespread and have been isolated from several thermal biotopes, including terrestrial hot springs in Colombia (2), Bolivia (3), and Italy (4); heated subsurface aquifers in Australia (5, 6) and India (7); and methanogenic sludge samples (8, 9). *Caloramator* spp. have attracted interest in the field of applied bioprocessing due to their robust growth on several carbohydrates derived from plant biomass while producing molecular hydrogen and ethanol at high yields (10–12).

In an effort to discover novel thermophilic, ethanol-producing microorganisms, environmental samples were collected within the Rabbit Creek area (44.52131°N, −110.81150°W) in Yellowstone National Park and maintained under anaerobic conditions (13). Using hot spring sediment and water as sources of inoculum, anoxic, thermophilic enrichment cultures were established using fermentative cellulolytic anaerobe (FCA) broth as a base medium (14), with milled switchgrass and Populus plants (0.1% [wt/vol]) as complex plant biomass substrates. Isolation attempts from the enrichments using a high-throughput, anoxic flow cytometry-based method described by Hamilton-Brehm et al. (13) yielded several isolates within the genus *Caloramator* that grew on cellobiose at 60°C. A rapidly growing strain that produced ethanol and acetate at a 2:1 ratio was designated *Caloramator* sp. strain E03 and selected for further analysis.

*Caloramator* sp. E03 was cultured at 60°C (pH 7.3), using 5 g/liter cellobiose in 50 ml of anaerobic FCA medium (14). Genomic DNA was purified from lysed cells using a phenol-chloroform method, followed by a final cleanup with the Genomic DNA Clean & Concentrator kit (Zymo Research, Irvine, CA). Long-read sequencing of genomic DNA for *Caloramator* sp. E03 was performed by the Department of Energy (DOE)’s Joint Genome Institute (JGI) on the PacBio RS II platform using a >10-kbp SMRTbell library (Pacific Biosciences, Menlo Park, CA, USA) (15). All general aspects of library construction and sequencing performed at the JGI can be found at http://www.jgi.doe.gov.

Sequencing resulted in 284,615 filtered subreads, which were reduced to 261,652 reads after additional filtering to remove reads of less than 1,000 bp. Filtered reads were assembled using Canu v1.5 to produce a draft genome of nine scaffolds (16). Manual gap filling and additional read mapping were performed by mapping the trimmed reads back to the scaffolds from the assembly using the “Map to Reference” option with 10% maximum mismatches per read, minimum mapping quality of 10, and otherwise default parameters. Regions where reads extended beyond the scaffold edges (coverage, >10×) were then added to the scaffolds in an iterative fashion until scaffolds were linked. Reads were then mapped back to the linked scaffolds to ensure that no variants or rearrangements were apparent. The assembly was reduced to a single scaffold, which was then circularized.

The single circular chromosome is 2,984,770 bp in length with a G+C content of 31.3%. Long-read data mapped back to the genome for over 220× coverage; the *L*_50_ value was 1 and the *N*_50_ value was 2,984,770 bp. Using VirSorter, the genome was searched for phage elements; three prophage regions were identified (17). The genome was annotated using the NCBI Prokaryotic Genome Annotation Pipeline and was predicted to contain 2,678 protein-coding sequences, 61 tRNAs, and 16 rRNAs in 3 operons (18).

### Data availability.

The 16S small-subunit ribosomal gene sequence GenBank accession number is HQ342687. The complete *Caloramator* sp. strain E03 genome sequence was deposited to GenBank under accession number CP040093. The raw sequence reads have been deposited in the Sequence Read Archive (SRA) under accession number PRJNA541090.
